# On Mechanical and Electrical Coupling Determination at Piezoelectric Harvester by Customized Algorithm Modeling and Measurable Properties

**DOI:** 10.3390/s22083080

**Published:** 2022-04-17

**Authors:** Irene Perez-Alfaro, Daniel Gil-Hernandez, Nieves Murillo, Carlos Bernal

**Affiliations:** 1 TECNALIA, Basque Research and Technology Alliance (BRTA), P° Mikeletegi 7, E-20009 Donostia-San Sebastian, Spain; daniel.gil@tecnalia.com; 2 Electronics Engineering and Communication Department, Universidad de Zaragoza, Pedro Cerbuna 12, E-50009 Zaragoza, Spain; cbernal@unizar.es

**Keywords:** energy harvesting, PZT, modeling, algorithm, harvester design, electromechanical, bimorph, electromechanical coupling

## Abstract

Piezoelectric harvesters use the actuation potential of the piezoelectric material to transform mechanical and vibrational energies into electrical power, scavenging energy from their environment. Few research has been focused on the development and understanding of the piezoelectric harvesters from the material themselves and the real piezoelectric and mechanical properties of the harvester. In the present work, the authors propose a behavior real model based on the experimentally measured electromechanical parameters of a homemade PZT bimorph harvester with the aim to predict its V_rms_ output. To adjust the harvester behavior, an iterative customized algorithm has been developed in order to adapt the electromechanical coupling coefficient, finding the relationship between the harvester actuator and generator behavior. It has been demonstrated that the harvester adapts its elongation and its piezoelectric coefficients combining the effect of the applied mechanical strain and the electrical behavior as a more realistic behavior due to the electromechanical nature of the material. The complex rms voltage output of the homemade bimorph harvester in the frequency domain has been successfully reproduced by the proposed model. The Behavior Real Model, BRM, developed could become a powerful tool for the design and manufacturing of a piezoelectric harvester based on its customized dimensions, configuration, and the piezoelectric properties of the smart materials.

## 1. Introduction

Power sources for autonomous IoT device developments, including sensing and communications means, are a current need towards the digitalization of society and, specificity, in the Smart Industry framework, where 5G will provide a full connectivity backdrop to accelerate self-powered IoT system penetration into the day-to-day of the factories and machines for their progress towards increasing productivity, reducing costs, and improving efficiency.

The main barrier for the massive deployment of IoT devices is their energy supply in a self-sufficient and self-autonomous manner to guarantee the convergence of the digital and physical worlds. Nowadays, autonomous devices are mainly battery-operated, with the consequent economic and environmental impacts. The increasing number of batteries used a year (800,000 tons of automotive batteries, 190,000 tons of industrial batteries, and 160,000 tons of consumer batteries in the European Union [1]), even if they are properly collected and recycled, has become an economical and an environmental problem to be solved due to the massive deployment of critical and consumer IoT devices, where critical IoT devices are those related to the management and the monitoring to supervise and control critical subsystems and equipment at smart manufacturing, infrastructures, and green energy.

Concepts such as Industry 4.0 or 5.0, Artificial Intelligence, Big Data, and Internet of Things have emerged to promote the current industrial and social revolution and are needed the support IoT devices, mainly centered on the use of intelligent sensors and systems for massive data acquisition, as the necessary link between the physical and virtual world, which can be implemented in any type of product or process [1,2,3]. However, this new generation of smart devices needs a continuous and autonomous energy source to guarantee a sustainable penetration of IoT technologies into the Smart Industry process. These demands, combined with the recent advances in ultra-low-power devices and electronics, has made energy harvesting, EH, technologies a powerful tool in realizing the scenario of self-powered autonomous IoT devices, where energy is harvested from environment sources, such as mechanicals, vibrations [4], thermal [5], sound, solar, and radio frequency [6] dissipated energies.

Energy harvesting is defined as the ability of smart materials to scavenge energy losses from sources operating in the surrounding environment for later use [7]. Those sources come from ambient environmental sources like thermal or temperature changes, gaseous or liquid flow fluctuations [8], electromagnetic radiations [9], chemical or physiological reactions [10], vibrational or acceleration movements, mechanical strain, or radiofrequency, among others [11]. The link between these sources and electrical power conversion are the smart materials, thermoelectric for thermal conversion, piezoelectric for mechanical, vibrational or movement, where the electromagnetic devices that can also scavenge environmental energy, photovoltaic for luminous emissions or triboelectric to harvest the electrostatic force, are the most relevant families of smart materials in the energy harvesting framework. Nevertheless, the piezoelectric materials present two main advantages, their high energy productivity and their power density that, combined with their wide frequency ranges, make them an essential tool for self-powered autonomous IoT device development. These unique properties make them a unique tool for EH solution development because of piezoelectric materials providing a consistent source of energy and the potential for electrical power generation. One of the most important factors to be considered is the need for a harvesting unit design based on a particular application—mainly, centred on the necessary levels of voltage or current required by the devices to be powered, adapted to the shape or size available of a specific application.

The design of piezoelectric harvesters is a relevant research topic, and different theories have been proposed for the development of IoT sensing and communication devices in a sustainable way [12]. One of the most successful concepts is the combination of multiple layers of piezoelectric materials with interlaminar conductive layers named bimorph or multi-morph, depending on the number of piezoelectric layers [6]. The interlaminar layer has mechanical and electrical functions, the last one depending on the desired electrical configuration, and it can be designed from several types of materials, from metallic to composites, among others. The bimorph or the multilayer harvester show higher energy density compared with a single piezoelectric unit when compared in the same vibrational states [7,8,9]. In general, research efforts to maximize energy scavenging of the harvester designs have been focused on the dimensional modification of the piezoelectric beam or the modification of their natural frequency by external mass additions.

Nevertheless, recent research into piezoelectric EH has emphasized the need to develop new models to optimize the design parameters of the harvester [13,14,15,16,17,18,19] and to adapt the electronic stage to minimize the electrical power losses of the devices [20,21]. Over the years, numerous circuits have been published with the aim of modeling piezoelectric-type generators, from the simplest ones where no losses are assumed and, therefore, no resistive parameter appears [22,23] to others more highly complex [24,25]. However, the most widely used circuit model is called the Butterworth van Dyke model [26,27], which consists of two circuit nettings separated by a transformer, the first of which refers to the mechanical parameters, while the second is defined by the electrical parameters domain. The Butterworth van Dyke model has been improved by some authors neglecting the losses of the dielectric layer [17]. However, there are few models in the bibliography based on the real parameters obtained from a harvester by experimentation. Due to this fact, the present work proposes a new behavior real model, BRM, for a piezoelectric harvester based on the one proposed by Butterworth van Dyke. The main difference of the proposed BRM and the Butterworth van Dyke one is that the BRM considers the electrical power losses during the harvesting process. The model has been formulated according to state of space equations, and it has been verified by the real harvester parameters obtained by experimental methods. These parameters of the piezoelectric bimorph actuator were tested experimentally aimed to determine the inertia moment, the damping behavior, and the stiffness coefficient. The use of experimental parameters allows a more realistic approach compared with the theoretical one. However, one necessary parameter has not been experimentally determined because of its complexity, the piezoelectric charge constant that represents the nexus between the mechanical strain and the electrical field. To solve the necessary input of the piezoelectric charge constant into the BRM model, an iterative customized iterative algorithm has been developed. This algorithm substitutes the complex finite element calculations [28] usually used for the piezoelectric constant determination. The experimental parameters focused on the dimensional changes of the harvester experimented due to the electric field and/or the mechanical strain, both applied and resulting from it because of the piezoelectric phenomena. As a result of the developed customized iterative algorithm, the values obtained for the piezoelectric load constant evidence some coefficient variations that could be explained in the framework of the internal adjustment of the piezoelectric materials due to the electrical field and mechanical strain presence. This piezoelectric coefficient variations might be associated with small behavior variations between the different piezoelectric coefficients, d_31_, d_33_, and d_15_, where a nonunique coefficient should be considered to represent the real harvester performance and where the bidirectionality of the piezoelectric effect could be the reason for the piezoelectric coefficient variation, since the mechanical deformation induces an electric field, but local variations of the electric field could induce small deformations too, affecting the piezoelectric charge constant behavior, and a nonunique constant should be considered for calculation of the maximized the energy harvesting of the piezoelectric bimorph.

## 2. Materials and Methods

### 2.1. Harvester Design and Preparation

The bimorph piezoelectric harvester has been designed and developed to obtain the experimental parameters necessary for the mathematical model. Nevertheless, a lot of research has been conducted in the energy harvesting of such a harvester to understand their experimental behavior. To guarantee reproducibility of the homemade specimens, the impedance behavior of the bimorph piezoelectric actuator has been measured as the footprint of the harvester. The PZT harvester has been electrically connected in a serial configuration, since this setting generates larger voltage figures compared with the parallel electrical configuration [29,30]. Electrically, a series refers to the case where the supply voltage is applied across all PZT beam layers at once.

**Figure 1:**
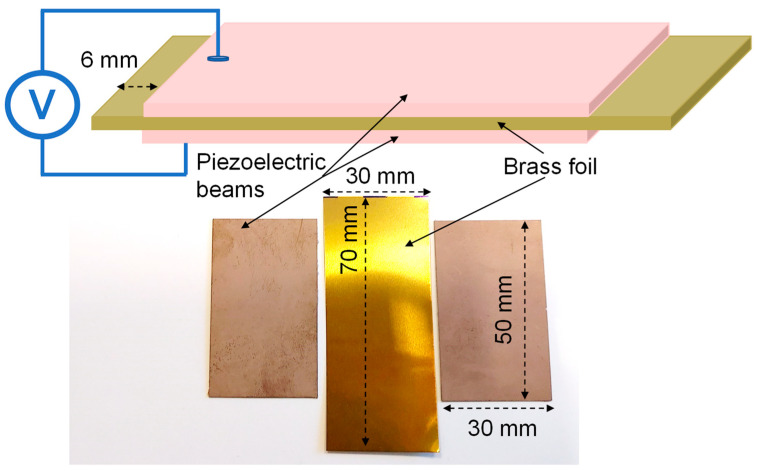
Bimorph piezoelectric harvester beam scheme and materials.

The bimorph PZT harvester structure is shown in Figure 1, where two PZT layers of Navy type II material are separated by a metallic layer of copper in beam configuration. The interlayer material was selected based on two required functionalities. The first one is the electrical conductivity between the PZT layer electrically connected into a series configuration. And the second is to provide the necessary mechanical rigidity to the overall bimorph structure. One of the most interesting materials that combines both functionalities is brass, widely use in acoustic applications [31]. Brass with a 125-µm = thick layer with a chemical composition of 63% copper and 37% zinc from Goodfellow Cambridge Limited was selected. The brass metallic layer is the interlaminar plane underlying both Navy type II PZT layers. Those were provided by PI Ceramic GmbH Keramische Technologien und Bauelemente with the PIC255 reference and dimensions of 0.2 × 50 × 30 mm^3^. The PIC255 PZT material was selected because of its high coupling factor and piezoelectric charge constant, both very appreciated for the actuators design in dynamic applications. The commune mechanical requirement of the energy harvester systems and the actuators based on piezoelectric is a low mechanical quality factor to guarantee the minimum energy lost per cycle, which is one of the PIC255 material characteristics. To join both PZT layers to the brass one, a conductive adhesive was applied. This allowed electrical charge conduction generated by the PZT dipole deformation between the piezoelectric ceramic layer and the metallic brass one. Finally, the harvester is electrically connected in the serial configuration. Both endings of the brass beam are PZT-free, see Figure 1, to hold the beam to the shaker during the testing experiments.

**Figure 2:**
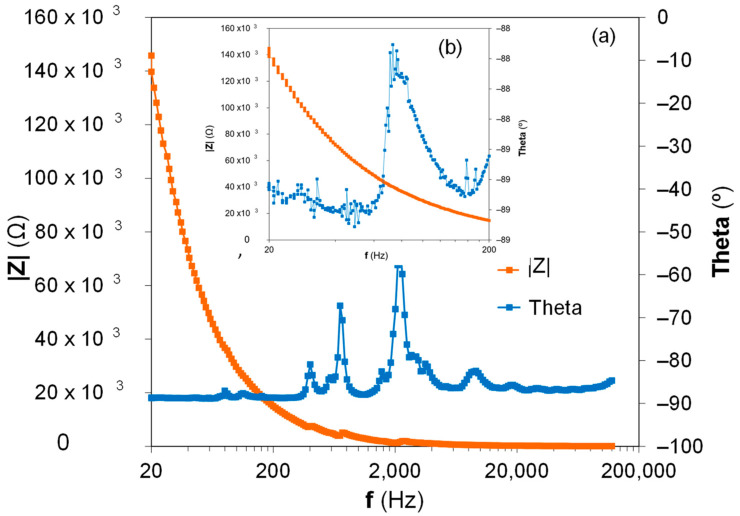
Impedance characterization in the frequency domain for the homemade PIC255 bimorph piezoelectric harvester: (**a**) frequency range from 20 Hz to 120 KHz and (**b**) frequency range from 20 Hz to 200 Hz.

The impedance characterization of the bimorph PZT harvester has been carried out as the footprint of the specimens. The impedance footprint of piezoelectric materials is the most efficient method to determine their optimal preparation due to ceramic weakness [32]. The presence of fissures at the PZT layer or the lack of electrical conductivity in the adhesive allows significant differences between the impedance spectrum of two similar samples. The equipment used to measure the impedance of the bimorph harvester was a LCR model E4980AL from Keysight Technologies with a frequency range between 20 Hz and 300 KHz. In Figure 2a, it can be seen that the impedance characterization is in a measured range from 20 Hz to 300 KHz. The insert of Figure 2b showed the detailed evolution of the impedance modulus and phase at the operational frequencies of the piezoelectric harvester from 20 Hz to 200 Hz. The impedance characterization of the full harvester is similar to that observed at the individual PIC255 PZT layers, reflecting their optimal piezoelectric properties performance and the lack of failures during their preparation.

### 2.2. Experimental Setup

**Figure 3:**
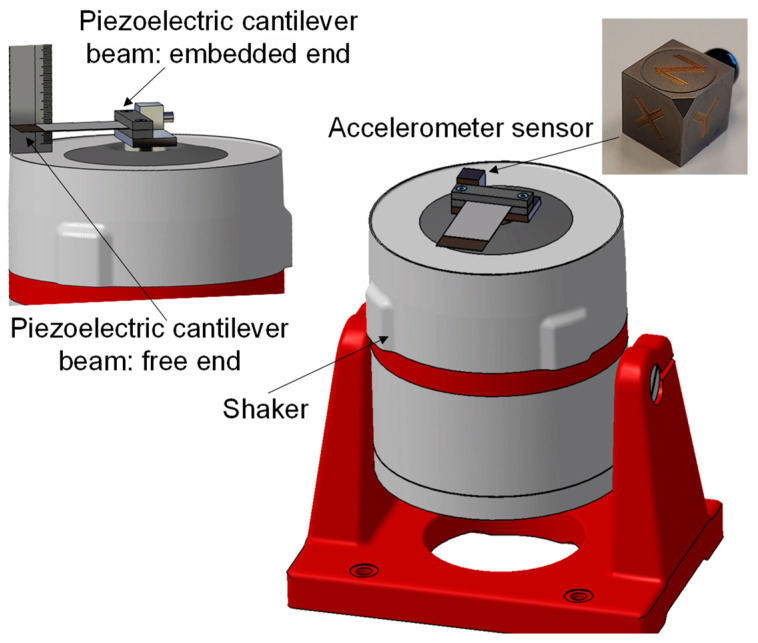
Schematic representation of the experimental setup for the measurement of the characteristic parameters of the piezoelectric bimorph harvester. The vibration system transmits a sinusoidal movement to the embedded end of the harvester at the selected acceleration. The amplitude and the frequency of the excitation signal were measured by an accelerometer sensor placed at the fixed end of the beam.

The mechanical properties used for the model evaluation were obtained based on the experimental setups described at the present section. The harvester was excited by shaker equipment, model LDS V406 from Hottinger Brüel & Kjaer Gmbh with an excitation acceleration of ±15 m/s^2^ for a frequency range between 15 Hz and 30 Hz. This applied vibrational force excited the bimorph piezoelectric harvester with a sinusoidal movement. The vibrational excitation energy was sufficient enough to achieve the maximum energy scavenging from the harvester, f_max_ = 22 Hz. To measure the movement features of the piezoelectric harvester at its fixed end, position A, an accelerometer sensor was used, model 356A15 from PCB Piezotronics, Inc. The bimorph harvester was fixed to the shaker as a beam, Figure 3, where the embedment end of the PZT harvester was named the fixed end or position A, and the free end of the beam was referred to as the free end or position B. The accelerometer was located at the fixed end of the PZT bimorph harvester. The accelerometer sensor measured the induced acceleration generated during the harvester vibrational excitation applied by the shaker. To achieve all the necessary movements characteristic of the bimorph cantilever, the speed and the position were calculated to form the acceleration integration. In order to measure the free end movement parameters, a high-speed camera was used, model RX100V from SONY Europe B.V. The free end movement of the piezoelectric harvester was recorded during shaker excitation. The position of the free end of the piezoelectric beam with respect to the time was determined as a correlation function of a slow-motion image analysis of the high-speed camera, and its derivate allowed to speed and acceleration the values of the free end of the piezoelectric bimorph harvester.

## 3. Model and Equivalences Proposition

### 3.1. Harvester Model Approach

**Figure 4:**
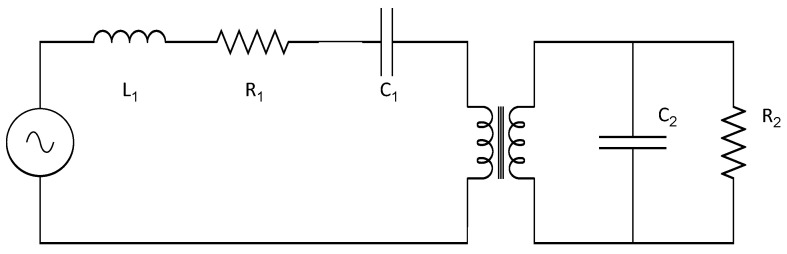
Butterworth Van Dyke equivalent circuit of a piezoelectric generator.

Butterworth Van Dyke [26,27], Figure 4, is considered as the grounding model in the electrical equivalence of the piezoelectric harvester behavior. The Butterworth Van Dyke equivalent circuit presents two separated circuits; both have been examined separately for their correlations with measurable values of the characteristic circuit components or of the bimorph piezoelectric harvester. This correlation will allow the proposition of the state of the space equation for the behavior real model proposition. The first part of the equivalent circuit regulates the mechanical behavior of the piezoelectric harvester represented as the inductance, L_1_, the resistance, R_1_, and the capacitance, C_1_, in the series configuration. The second one is the equivalence with the electrical behavior regulation, and it is represented as a circuit consisting of a resistance parallel to the capacitance, named C_2_ and R_2_. Both equivalent circuits, representing the mechanical and the electrical behavior, are coupled by an electrical transformer. To estimate the mechanical equivalent circuit, the parameters that govern the behavior of a solid movement are its damping, the exposed inertial forces, and its stiffness coefficients [33,34], and they are represented in the mechanical equivalent circuit as L_1_, C_1_, and R_1_. On the other hand, the electrical equivalent circuit parameters, L_2_ and C_2_, could be experimentally determined by LCR meter measurements.

Finally, the coupling between both equivalent circuits, the electrical and the mechanical, represented at the Butterworth Van Dyke by the electrical transformer, was correlated with the piezoelectric charge constant, since this coefficient defines the deformation induced in the piezoelectric harvester by an applied electrical field. Otherwise, the piezoelectric constant is the link between the mechanical and the electrical variables affecting the harvester behavior under specific applied conditions.

To propose a behavior real model based on measurable properties during the harvester functioning, it is necessary to stablish equivalences between the electrical and the mechanical properties of the bimorph harvester based on the Butterworth Van Dyke equivalent circuits. The equivalences are summarized at Table 1, where the correlation between the electrical equivalent elements of the Butterworth Van Dyke model and the real measurable properties, electrical and mechanical, linked to them are defined. These equivalences are based on two considerations: the first one is that the energy generated by the piezoelectric harvesters is considered the result of the charge movements between the PZT dipoles, and the second one is that the equivalences are proposed in terms of the work exchanged between the electrical and mechanical functioning of the harvester. In Table 2, the dimensional equivalences of the magnitudes of Table 1 are shown.

The bimorph piezoelectric harvester is analyzed in its vibrational configuration as a solid cantilever beam with its one end fixed in order to utilize the flexural mode of the structure for the energy conversion from the vibrational forces. The PZT bimorph harvester prepared as result of three layers, two PIC255 PZT and one brass layer, is considered as a unique solid from the electrical and mechanical points of view, where the equations that govern the behavior of rigid solids are considered valid, although the free end of the bimorph beam undergoes greater elongation during vibratory movements than the fixed one.

### 3.2. Equivalences between the Electrical and the Mechanical Circuits

The equivalences where the link between the electrical properties and the mechanical ones proposed in Table 1 are sustained in the hypothesis and dimensional analysis detailed in this section, where the equivalences between the electrical work measured at the harvester and the mechanical measurable properties of the PZT bimorph harvester allow a model development based on the state of the space equations named the Behavior Real Model.

1. Stiffness—Stiffness (electrically equivalent to C_1_) was defined as the ratio between the force applied on a system and the displacement it causes. Therefore, the stiffness coefficient could be determined by dividing the applied force on the piezoelectric beam by the difference between the displacement of the free end, $x_{B}$, and the displacement of the fixed one, $x_{A}$.
  (1) $$Stiffnes=\frac{\left|F_{applied}\right|}{\left|x_{B}-x_{A}\right|}\;≅\;\frac{1}{C_{1}}$$

2 Damping—The damping coefficient (electrically equivalent to R_1_) was defined as the ratio between the applied force on a system and the speed it acquires. The experimental determination of the damping coefficient of the piezoelectric bimorph harvester at its fixed end was calculated using the integral of the acceleration measured by the accelerometer sensor, $v_{A}$. The speed of the free end,${\;v}_{B}$, was obtained from the function determined by the high-speed camera measurements and its subsequent derivation.
  (2) $$Damping=\frac{\left|F_{applied}\right|}{\left|v_{B}-{\;v}_{A}\right|}≅R_{1}$$

3 Inertia—The inertia that governs the behavior of the PZT bimorph harvester was analyzed for the electrical equivalence, the inductance, to determine the mechanical properties that could be experimentally measured as the input for the model. The electrical behavior of an inductance is governed ideally by the following equation [35]:

(3) $$I\;=\frac{1}{L_{1}}\;\int^{\;}V\cdot \;\mathrm{dt}$$

where V is the electrical voltage, and I is the electrical current of the inductance.

The inductance is usually modeled in piezoelectric harvesters by the Finite Element Method, FEM. Nevertheless, in the present work, all the equivalences were performed approaching the equivalences between the electrical and the mechanical behaviors of the piezoelectric bimorph carried out by the harvester. That means the equivalence between the electrical work was delivered by the harvester and the mechanical work induced by the vibrational forces. In this sense, the equivalence between the mechanical behavior of the inductance was proposed based in the following hypothesis: the equivalence between the electrical and the mechanical forces in the modulus and the dimensional approach between the electrical density and the velocity are based on the dimensional equivalence between the damping and electrical resistance. This hypothesis proposition is based on the piezoelectric physical principle, where the electrical potential of a piezoelectric harvester is proportional to the applied force in terms of the work exchange between both magnitudes, and the second part of the hypothesis, where the equivalence between the electrical current and the resulting velocity of the harvester is proposed. This hypothesis is based on the dipole charge generation of the piezoelectric materials resulting from the speed experimented by the harvester due to their vibrational conditions, which are responsible for the electrical current density of the bimorph. Appling this hypothesis to Equation (3), the inductance could be expressed as follows:

(4) $$L_{1}=\frac{\int^{\;}V\;\mathrm{dt}}{I}\approx \frac{\int^{\;}\left|F_{m}\right|\;\mathrm{dt}}{v_{r}}$$

where, $F_{m}$ is the applied mechanical force, and $v_{r}$ is the resulting speed.

Taking Equation (3) in increments, the resulting equation is:

(5) $$\Delta L_{1}=\frac{\left|F_{m}\right|}{a_{m}}=\;m\cdot \frac{a_{p}}{a_{r}}$$

where $a_{r}$ is the resulting acceleration, $a_{p},$ is the applied acceleration, and m, is the harvester mass.

The resulting Equation (5) is expressed in experimentally measurable properties of the harvester as consequence of the vibrational force application.

4 The piezoelectric charge constant, usually named *d*, is the link between the mechanical strain produced by the applied electrical field when the piezoelectric material acts as an actuator. Conversely, this coefficient may be assigned to the resulting electrical charges collected by the harvester electrodes when mechanical stress is applied. The piezoelectric charge coefficient directions depend on the direction of the applied force and the polarization of the piezoelectric layer, $d_{ij}$. In the present model, the equivalence between the piezoelectric charge constant, mechanical property, and their electrical equivalence in the Butterworth Van Dyke model is named N, as defined in Table 1. This coefficient, N, connects and governs the electrical or mechanical forces of the bimorph piezoelectric harvester. The dimensional units of the piezoelectric coefficient are [C/N], which could be expressed as [A·s/N ]. The dimensional equivalence in the mechanical circuit is [m/V]. This coefficient is characteristic of piezoelectric generators, but it is not a property of a conventional electrical transformer. The conventional transformer acts by reducing or increasing the electrical voltage between both networks at the Butterworth Van Dyke equivalent circuits, while the piezoelectric generator acts by linking the electrical voltage of one equivalent circuit with the integral of the current intensity by the differential of the time. The dimensional equivalences are shown in Table 3.

### 3.3. Behavior Real Model Based on State of Space Equations

The equivalences between the electrical and mechanical parameters of the piezoelectric bimorph harvester were proposed in the previous section based on the Butterworth Van Dyke equivalent model, Figure 4 and Table 1, and they will be used for the BRM development, according to the Kirchhoff mesh law. The electrical potential difference in the left mesh of the circuit that model the piezoelectric harvester behavior, Figure 4, could be defined as follows:

(6) $$\left|F\right|\propto L_{1}\cdot \frac{{\mathrm{dI}}_{1}}{\mathrm{dt}}+R_{1}\cdot I_{1}+\frac{1}{C_{1}}\int^{\;}I_{1}\;\mathrm{dt}+V_{1}$$

where $I_{1}$ is the electrical density at the left mesh of the Butterworth Van Dyke equivalent circuit, is the measurable voltage at the primary coil of the transformer, and $C_{1}$ and $L_{1}$ the capacitance and inductance, respectively, corresponding with the previously mentioned equivalent circuit.

The transformer associated with both equivalent circuits and the relationship between the electrical potential difference of the primary circuit and the current density of the secondary equivalent circuit, the right circuit in Figure 4, could be written as shown in the last term of Equation (7), considering N as the piezoelectric generator magnitude to connect the electrical and mechanical behaviors. Introducing this consideration into Equation (6), |F| could be written as:

(7) $$\left|F\right|\propto L_{1}\cdot \frac{{\mathrm{dI}}_{1}}{\mathrm{dt}}+R_{1}\cdot I_{1}+\frac{1}{C_{1}}\int^{\;}I_{1}\;\mathrm{dt}+\frac{1}{N}\int^{\;}I_{2}$$

The electrical current density in the second mesh, the electrical parameters in the circuit of Figure 4, is described as follows:

(8) $$I_{2}={\;C}_{2}\cdot \frac{{\mathrm{dV}}_{2}}{\mathrm{dt}}+\frac{V_{2}}{R_{2}}$$

The combination of both equation results is obtained:

(9) $$\left|F\right|={\;L}_{1}\cdot \frac{{\mathrm{dI}}_{1}}{\mathrm{dt}}+R_{1}\cdot I_{1}+\frac{1}{C_{1}}\int^{\;}I_{1}\;\mathrm{dt}+\frac{1}{N}\left(\int^{\;}C_{2}\cdot \frac{{\mathrm{dV}}_{2}}{\mathrm{dt}}\mathrm{dt}+\int^{\;}\frac{V_{2}}{R_{2}}\mathrm{dt}\right)$$

(10) $$\left|F\right|={\;L}_{1}\cdot \frac{{\mathrm{dI}}_{1}}{\mathrm{dt}}+R_{1}\cdot I_{1}+\frac{1}{C_{1}}\int^{\;}I_{1}\;\mathrm{dt}+\frac{C_{2}}{N}\cdot V_{2}+\frac{1}{N\cdot R_{2}}\cdot \int^{\;}V_{2}\;\mathrm{dt}$$

Clearing I_1_ from the previous equation, it is obtained:

(11) $$\frac{{\mathrm{dI}}_{1}}{\mathrm{dt}}=\frac{\left|F\right|}{L_{1}}-\frac{I_{1}\cdot R_{1}}{L_{1}}-\frac{1}{C_{1}\cdot L_{1}}\int^{\;}I_{1}\;\mathrm{dt}-\left(\frac{1}{N\cdot R_{2}\cdot L_{1}}\right)\int^{\;}V_{2}\mathrm{dt}\;-\frac{C_{2}}{N\cdot L_{1}}\cdot V_{2}$$

Taking again into account the relationship between the secondary circuit voltage, right equivalent circuit of Figure 4, and the current density of the primary circuit, the left equivalent circuit in the figure, through parameter N, is obtained:

(12) $$V_{2}\cdot N\;=\int^{\;}I_{1}\;\mathrm{dt}\to \frac{{\mathrm{dV}}_{2}}{\mathrm{dt}}=\frac{I_{1}}{N}$$

where $V_{2}$ is the voltage measured in the secondary winding of the transformer.

These equations are used for the matrix proposition to the state of the space model, which is governed by an output voltage $V_{2}$, generated through an input force modulus |F|:

(13) $$\left[\begin{array}{c} \dot{I_{1}}\\ \dot{V_{2}}\\ I_{1}\\ V_{2} \end{array}\right]=\left[\begin{array}{cccc} -\frac{R_{1}}{L_{1}} & -\frac{C_{2}}{N\cdot L_{1}} & -\frac{1}{C_{1}\cdot L_{1}} & -\frac{1}{N\cdot R_{2}\cdot L_{1}}\\ \frac{1}{N} & 0 & 0 & 0\\ 1 & 0 & 0 & 0\\ 0 & 1 & 0 & 0 \end{array}\right]\cdot \left[\begin{array}{c} I_{1}\\ V_{2}\\ \int^{\;}I_{1}\\ \int^{\;}V_{2} \end{array}\right]+\left[\begin{array}{c} \frac{1}{L_{1}}\\ 0\\ 0\\ 0 \end{array}\right]\cdot \left[\left|F\right|\right]$$

(14) $$\left[V_{2}\right]=\left[\begin{array}{cccc} 0 & 1 & 0 & 0 \end{array}\right]\cdot \left[\begin{array}{c} I_{1}\\ V_{2}\\ \int^{\;}I_{1}\\ \int^{\;}V_{2} \end{array}\right]$$

These equations are used for the electrical behavior determination of the bimorph piezoelectric harvester, and the results are presented in the following sections.

## 4. Results

The experimental characteristic parameters for BRM model verification and the obtention of the electrical characteristics, L_1_, C_1_, R_1_, and N, of the bimorph piezoelectric harvester are presented in the present section.

**Figure 5:**
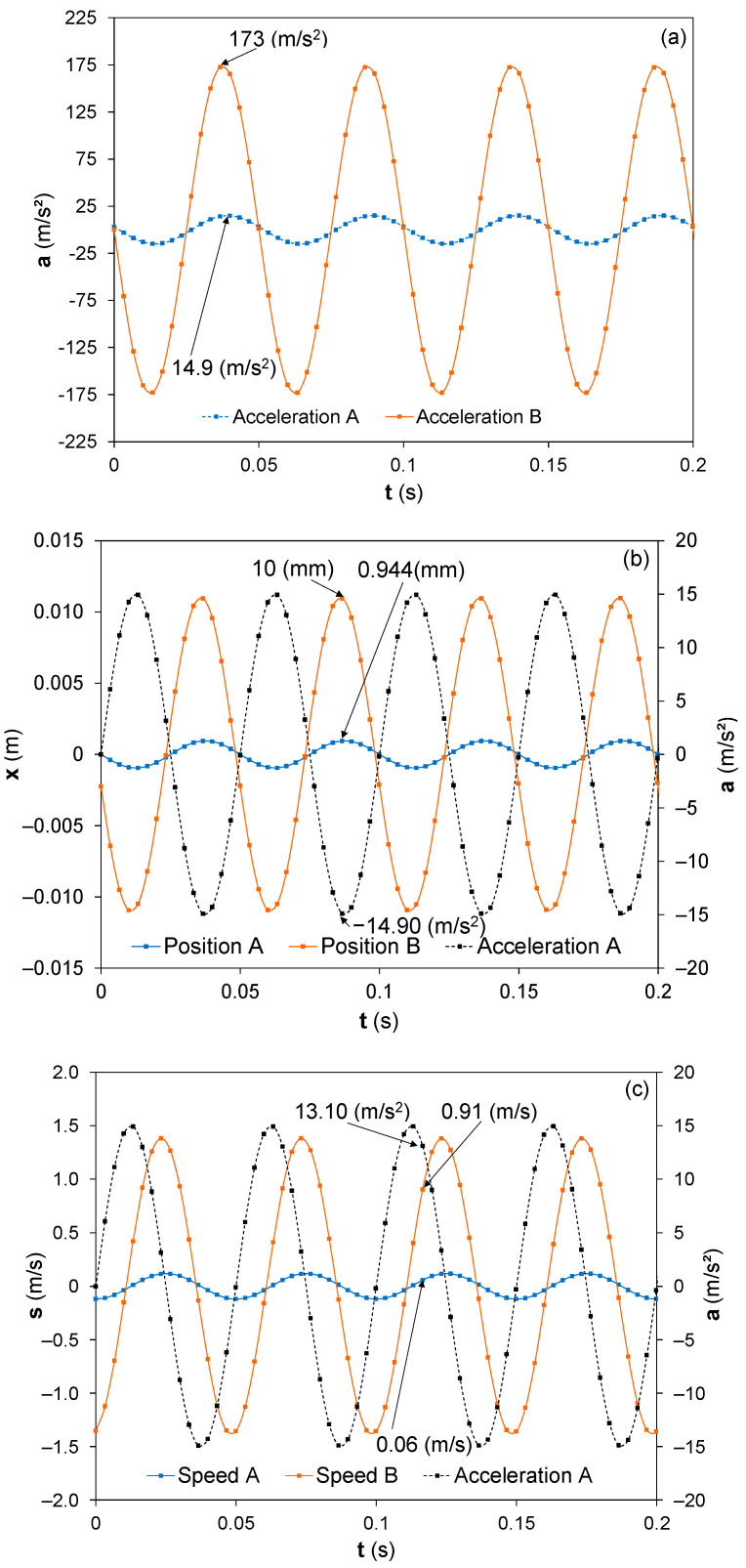
Experimental results of the piezoelectric bimorph cantilever at f = 20 Hz and excitation acceleration of ±15 m/s^2^. (**a**) The acceleration measurements at the free and the fixed ends of the beam, (**b**) the displacement obtained at both ends, and (**c**) the speed of the free end and the speed and the acceleration of the fixed end.

The experimental setup was described in Section 2.2, and the operational frequencies with the electrical, physical, and mechanical properties of the bimorph piezoelectric harvester were measured. The frequency range used in the present study was from 15 Hz to 30 Hz because of the maximum energy extraction of the bimorph homemade harvester occurring at this frequency range. The acceleration measured in the experimental setup, Figure 3, at the fixed end of the bimorph piezoelectric beams, named point A, and at the free end, named point B, is presented in Figure 5a. The images from a slow-motion camera show the synchronous movements between both ends of the bimorph harvester, the fixed and free ends. Nevertheless, to have a realistic comparison, it is necessary to identify the acceleration of both ends of the harvester at the same instant of time. There is only one specific point at Figure 5a where the acceleration of both ends of the harvester occur at the same instant of time, where the acceleration of A, the fixed point, was $a_{A}=14.9\;m/s^{2}$, and that of B, the free point, was $a_{B}=173\;m/s^{2}$.

The positions of the fixed and free ends were identified from the data represented in Figure 5b. The identification of the positions for both ends that occurred at the same temporal point was more difficult than in the case of acceleration. To identify this point, it was necessary to use the acceleration measured in the fixed end based on its acceleration measurements. The position of the free end, B, and fixed end, A, should be identified from the maximum displacement measured at both ends. At the fixed end, A, the point to be considered is that occurring at the time value where the acceleration exhibits its minimum value, since the acceleration at point A and its displacement are in opposite phases due to its mathematical relationship, $a\;\left(t\right)={\;d}^{2}\cdot r/{\mathrm{dt}}^{2}$. The experimental values obtained for the same temporal value, where $x_{A}=0.944\;\mathrm{mm}$ for the fixed end and $x_{B}=10\;\mathrm{mm}$ for the free end with an acceleration of $a_{A}=14.90\;m/s^{2}$ at the embedded end of the piezoelectric harvester cantilever.

Finally, it is necessary to determine the speed of the bimorph beam at both ends to complete the necessary experimental data for the model. It was necessary to make an approximation for both measurement and synchronization, because at the maximum acceleration, the speed is zero. Due to this fact, the speed values are those with the maximum error amount of the acceleration and the position. The approximation for synchronizing the speed and acceleration measurements was to consider that the speeds at both ends of the piezoelectric cantilever are in phase. The difference of the phase between the speed and the acceleration at the fixed end of the beam could be known due to its mathematical relationship, a=dv/dt. The observed speed at point A for an acceleration of $a=13.10\;m/s^{2}$ was v=0.06 m/s, where the speed at the free end observed was v=0.944 m/s. The measurements were carried out in a frequency range from 15 Hz to 30 Hz, and all the characteristic parameters obtained for the Behavior Real Model, BRM, are shown at Table 4.

Once the mechanical characteristic parameters, acceleration, speed, and position of the harvester were determined for the BRM evaluation, the electrical properties of the piezoelectric bimorph, C_1_, R_1_, and L_1_, based on the Section 3.2 equivalences were calculated. The values obtained for the three parameters, capacitance, resistance, and inductance, at the different excitation frequencies, from 15 Hz to 30 Hz, are detailed in Table 5.

In the classical electrical approach, a capacitance is related to the energy storage potential. In the case of the bimorph piezoelectric harvester, the energy generated by the induced sinusoidal movement is not stored at a capacitor properly, as in a classical electrical approach. The capacitance has its equivalence on the applied force per displacement unit, where the value of the piezoelectric force could be considered as a negative or positive load. J. Przybylski et al. [36] modeled the influence of the electric field on an axial compressive load at the natural frequency of a beam with a bounded piezoelectric patch using $f\;=\pm 1.4p_{C}^{\infty }$, where $p_{C}^{\infty }$ is the load and f the piezoelectric force for the actuators. The electrical force, when $f\;=-1.4p_{C}^{\infty }$ is applied, results in two higher ice forces than in the case of the positives ones. The negative capacitance obtained as a result of the BRM model based on electromechanical equivalences could be assigned to the occurrence of negative piezoelectric forces as a consequence of the electron transference between the dipoles due to the vibrational excitation of the piezoelectric bimorph harvester around the resonance frequency.

Furthermore, the electrical characteristic parameters of the right equivalent circuit at the Butterworth Van Dyke were measured under statics conditions. The measurements during the dynamic vibrational excitation of the piezoelectric harvester allow the values of C_2_ and R_2_, where the component of the electrical piezoelectric force of the mechanical equivalent circuit, left equivalent circuit in the Butterworth Van Dyke model, and component of the electrical variables of the electrical equivalent circuit, the right equivalent circuit of the Butterworth Van Dyke model, will be measured together. The internal resistance of the measurement equipment will also contribute. To reflect the real electrical characteristic parameters range, the measurements were performed under statics conditions for the working frequency range, from 15 Hz to 30 Hz. C_2_ and R_2_ were measured by a LCR with a configuration parallel resistance—capacitance equivalent circuit of the piezoelectric bimorph harvester. The capacitance obtained was C_2_ = 59 nF, and the R_2_ function was $R_{2}=-1.9\times 10^{5}\cdot f+9.55\times 10^{6}$. The R_2_ values are expected to increase the BRM model error, because a linear behavior is obtained due to it being measured under static conditions.

## 5. Discussion

The Behavior Real Model results based on the mechanical characteristic parameters measured experimentally are discussed at this section based on the electromechanical equivalences established from the Butterworth Van Dyke equivalent circuit. The evaluation of N,∝ Piezoelectric charge coefficient, was achieved based on a customized iterative algorithm, since non-direct measurements of the piezoelectric coefficients for the bimorph harvester during their vibrational excitation status could be done. After the N determination, a comparison between the V_rms_ output obtained theoretically and experimentally is presented at this section.

### 5.1. Electrical Characteristic Parameter from BRM

**Figure 6:**
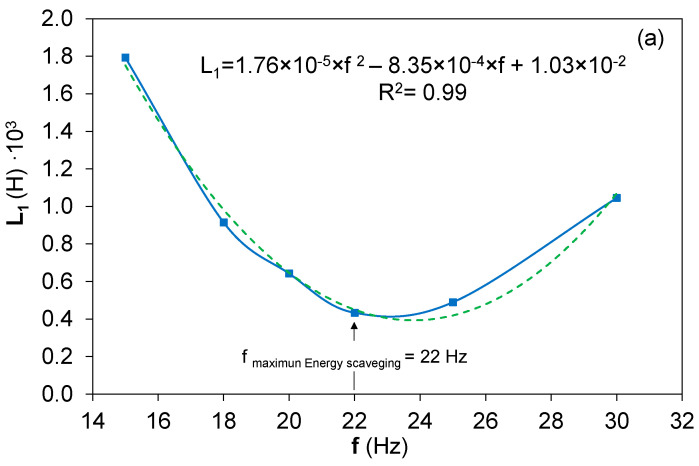

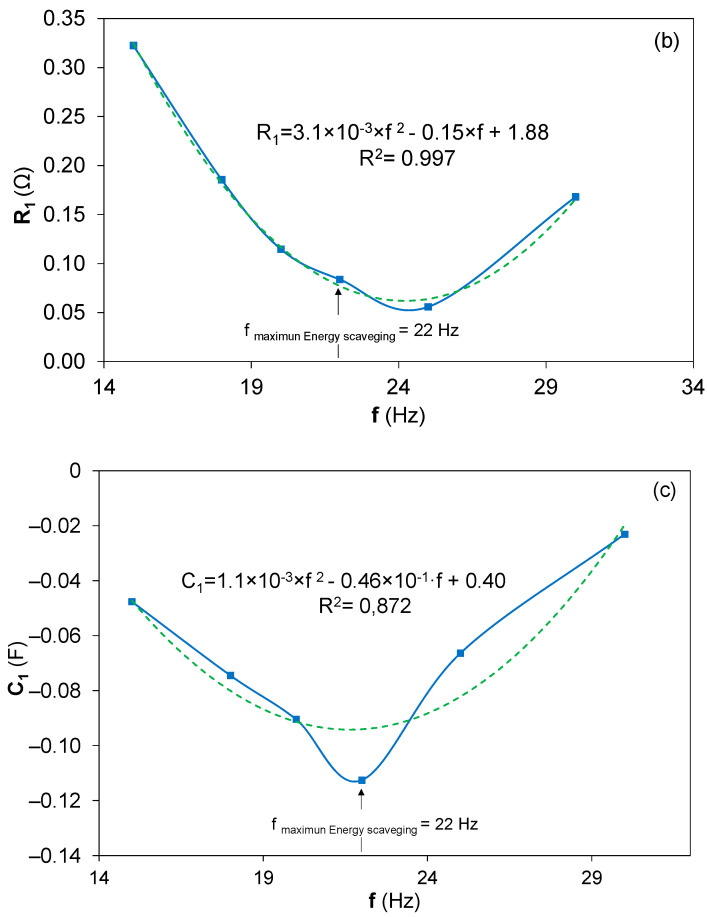
Electrical equivalences of (**a**) the inductance, L_1_, (**b**) the resistance, R_1_, and (**c**) the capacitance, C_1_, resulting from the BRM model based on the mechanical experimental results and their fitting into the second-order equation.

The electrical parameters, inductance, L_1_, capacitance, C_1_, and resistance, R_1_, obtained by the Behavior Real Model based on the electrical equation of the state and the measured acceleration, speed, and position from the mechanical sinusoidal excitation of the bimorph harvester are presented in Figure 6 as the function of the excitation frequency and an applied acceleration of ±15 $m/s^{2}$. The maximum energy scavenged from the bimorph piezoelectric harvester corresponded with a frequency of 22 Hz.

All the electrical characteristic parameters showed a nonlinear behavior, with a minimum value at the frequency where the maximum energy was harvested. 

The inductance decreased from 1.8 × 10^−3^ H until a minimum of 0.43 × 10^−3^ H around the frequency where the maximum energy was scavenged by the bimorph harvester, Figure 6a. This behavior could be understood as the accommodation of the electrical force to the mechanical changes caused by the vibrational loads of the piezoelectric beams. Due to the electromechanical coupling between the electrical and mechanical properties of the bimorph piezoelectric harvester, the inductance decreased to guarantee an increase of the dipole movement and the electrical charge flow between them. As a consequence, an electric potential was generated at the series bimorph harvester. The inductance continued, allowing higher values due to the increase of the inertia in the electric charge flow movement, and as consequence, the energy or the charge flow between dipoles decreased considerably. Moreover, the nonlinear behavior of the resistance could be the consequence of a small phase change between the inertia, represented by the inductance as the mechanical equivalence at the Butterworth Van Dyke circuit and the resistance associated with piezoelectric damping. Likewise, the capacitance linked with the stiffness or the elastic behavior of the bimorph piezoelectric around the resonance frequency decreased more drastically at this frequency, f = 22 Hz. This C_1_ characteristic parameter of the BRM model reflected the dielectric and elastic behaviors of the piezoelectric harvester as a consequence of the intrinsic internal phenomena occurring at the piezoelectric material due to the applied load, where the dipole movement resulted in their wall movement and reorientation due to the piezoelectric force of the applied load [37]. The inhomogeneities in the dominion distribution and the electrical field consequence of the applied load are optimized at the resonance frequency for the energy harvesting maximization.

### 5.2. Custom Iterative Algorithm for N Calculation

**Figure 7:**
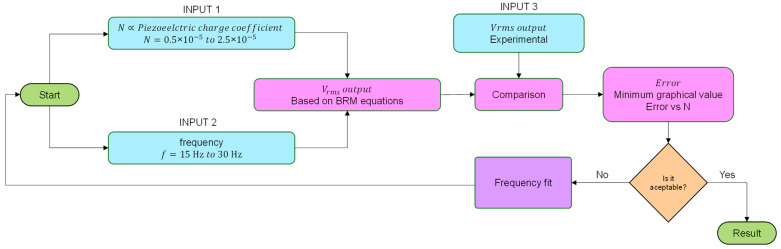
Scheme of the custom iterative algorithm.

The N coefficient, equivalent to the piezoelectric charge constant, was estimated by a customized iterative algorithm, because it was not possible to measure this experimentally during the sinusoidal load applications. The piezoelectric charge constant is a characteristic parameter of the PZT material provided by the manufacturer as an average value. The PIC 255 used in this work had the following *d_ij_* values: (a) *d_3_*_1_ = −180 × 10^−12^ C/m, (b) *d*_33_ = 400 × 10^−12^ C/m, and (c) *d*_15_ = 550 × 10^−12^ C/m. Moreover, the piezoelectric harvester had a bimorph configuration with a series connection, and the piezoelectric charge constant during the sinusoidal load application could modify itself, as it happened with the mechanical characteristic properties L_1_, R_2_, and C_2_ at the electrical equivalences obtained from the BRM model, Figure 6, because of the electromechanical coupling and the piezoelectric forces. Usually, a finite element model is used for this coefficient estimation [38]; nevertheless, in the present work, a customized iterative algorithm was developed to predict N. The iterative algorithm worked as comparative calculations between the measured output voltage of the generator and the output voltage estimated by the BRM model for a specific frequency when the vibrational load is applied, where the iterations are defined to achieve the maximum fitting concordance between the V_rms_ output voltage experimentally measured and the theoretical one, and the fitting ≈ 97.07%, as described in Figure 7.

**Figure 8:**
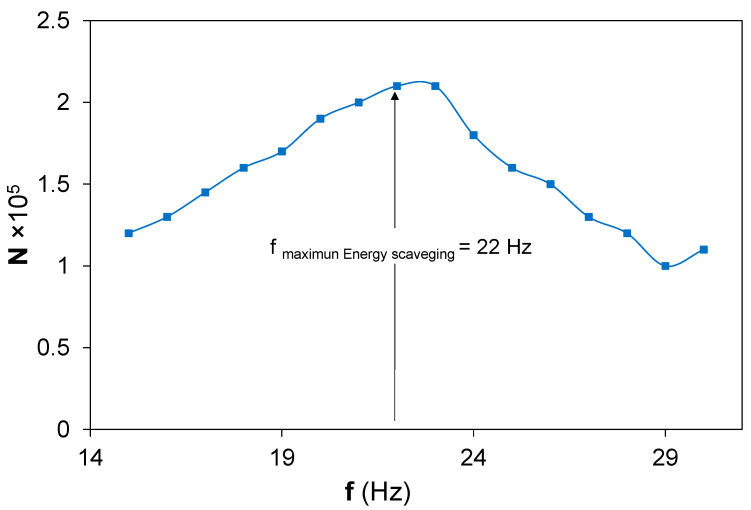
N coefficient calculated from the customized iterative algorithm versus frequency.

The N coefficient presents a nonlinear behavior with the frequency, with the maximum at the optimal energy scavenging frequency, Figure 8. This phenomenon could be understood as caused by dimensional changes of the bimorph piezoelectric harvester during sinusoidal load excitation as a consequence of the piezoelectric forces present at the bimorph beam [39]. The presence of small changes of the vibrational modes at the piezoelectric bimorph beam could affect the piezoelectric forces, resulting in different wall movements of the dipoles and their orientation under the electrical field produced by the vibrational loads.

However, the value obtained from the customized iterative algorithm for the N coefficient was proportional to 10^−5^ instead of the *d_ij_* ∝ × $10^{-12}$ exhibited by the piezoelectric charge coefficient. The reason was that no direct correlation between both coefficients was proposed at the BRM. The correlation between N and *d_ij_* could be done based on elongation phenomena due the electrical force that occurred when the piezoelectric bimorph harvester was sinusoidally excited due to the electromechanical coupling between both [40]. The static displacement, Δh, that took place when the piezoelectric material was under the effects of an electric field could be written as follows, ec. 15:

(15) $$\Delta h\;=\frac{3d_{31}\cdot V\cdot l^{2}}{2h^{2}}$$

where, Δh is the static displacement, $d_{31}$ is the piezoelectric charge constant, V the voltage used to fuel the piezoelectric actuator, l the piezoelectric length, and h the piezoelectric thickness.

The piezoelectric charge coefficient, *d_ij_*, based on the static displacement, Δh, equivalent to the N coefficient, N ≈ Δh, could be calculated based in the bimorph cantilever dimensions and the V_rms_ output, following Equation (16):

(16) $$d_{31}=\frac{\Delta h\cdot 2h^{2}}{3V\cdot l^{2}}=\;\Delta h\;\cdot \;\frac{2h^{2}}{3V\cdot l^{2}}=N\cdot \;\frac{2h^{2}}{3V\cdot l^{2}}$$

**Figure 9:**
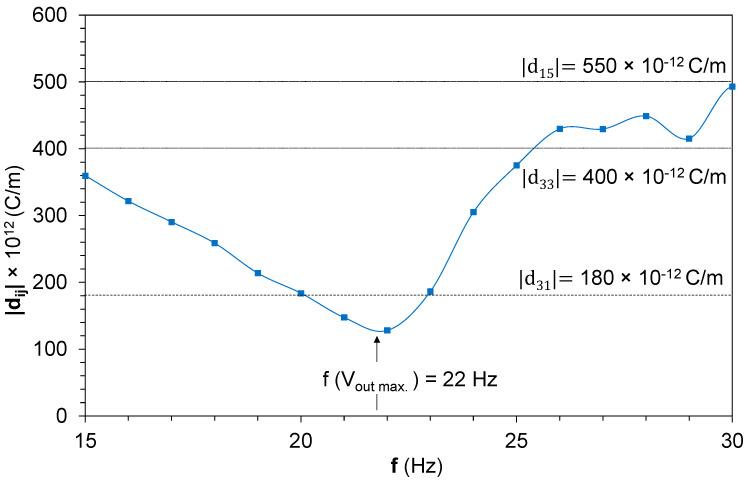
Theoretical *d_ij_* piezoelectric coefficient modulus versus the frequency of excitation.

The results from Equation (17) and the BRM characteristic parameters based on the N coefficient obtained from the customized iterative algorithm are shown in Figure 9, where *d_ij_* as the frequency function is charted. All the *d_ij_* coefficients are considered in its modulus values, because not polarization or the sinusoidal movement vector in the electrical or mechanical forces were considered in the BRM model due to the fact that the BRM model is focused on the electrical and mechanical works that the piezoelectric bimorph are is able to do during its vibrational excitation, and vectorial loses were not considered.

The *d_ij_* resulting from Equation (17), based on the BRM model and customized iterative algorithm, exhibits a complex behavior, where the governing piezoelectric charge constant at around the resonance frequencies is d_31_. The piezoelectric forces that the bimorph harvester undergo are pointed in the polarization direction, whereby the dipoles and the wall orientation move to the surface direction for the maximum electrons collection under the piezoelectric electrodes, and the forces are applied with the right angel to the polarization axis, resulting in d_31_. The governing piezoelectric charge coefficient, out of the resonance frequency ranges, is a combination of d_31_ with d_33_. This result could be understood as the dimensional changes affecting the volume of the piezoelectric bimorph harvester due to the piezoelectric forces induced by sinusoidal excitation. The volume changes could affect the wall movements of the dipoles, resulting in lower charges transference to the piezoelectric electrodes and, as consequence, a lower energy harvesting. These dipoles and wall movements due to the electromechanical force excitation of the piezoelectric material allow bimorph harvester adaptation to maximize the V_rms_ output. These understandings of *d_ij_* behavior under dynamic conditions will enable the energy scavenging maximization through the design optimization of the piezoelectric harvester based on dimensional characteristics and polarization properties of the harvester and the piezoelectric materials used for their manufacturing.

### 5.3. V_rms_ Outpout, Experimental and Theoretical Results

**Figure 10:**
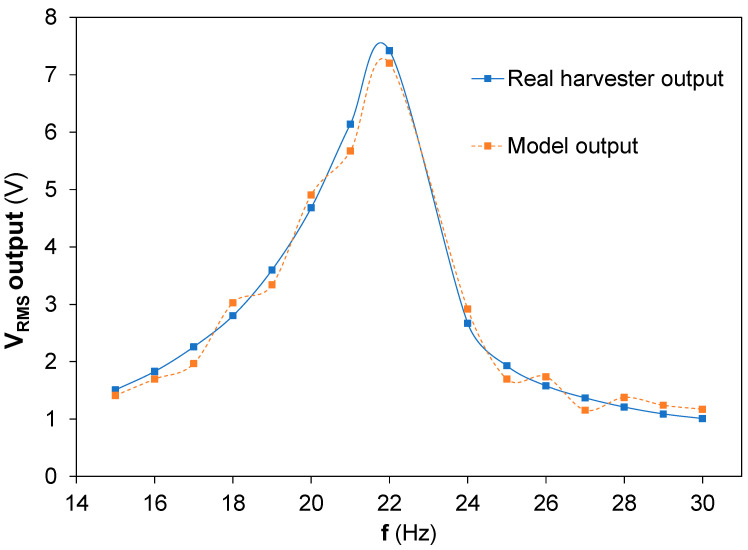
V_rms_ output experimental and theoretical versus frequency.

The V_rms_ output experimental and theoretical from the BRM model and the customized iterative algorithm are shown at Figure 10, where the frequency range goes from 15 Hz and 30 Hz at an applied acceleration amplitude of ±15 $m/s^{2}$. The theoretical values obtained from the BRM model present a similar behavior to those measured experimentally.

The accuracy between both results, experimental and theoretical, allows to conclude that a realistic model, Behavioural Real Model, based on the mechanical property measurements proposed for the equivalences established from the Butterworth Van Dyke equivalent circuits proposed a realistic approach to understand the bimorph piezoelectric harvester behavior. The direct mechanical characteristic parameter measurements, although experimental errors are present at the dynamic measurements of the angular acceleration, speed, and position, a minimal influence on the BRM theoretical results was noticed.

## 6. Conclusions

The main aim of this research work was focused on the understanding of piezoelectric harvester behavior under dynamic conditions in energy to maximize the harvesting potential under vibrational conditions. The most frequent approach to piezoelectric harvester modeling is based on the use of a combination between dynamic and constant properties. Few efforts have been done to the understand the dynamic behaviors of the electrical properties, mainly because of the difficulties in realistic property measurements without parasitic interferences of the measurement equipment. The proposition of the measurable characteristic parameters of the bimorph harvester based on Butterworth Van Dyke model equivalences resulted in a very accurate determination of the electrical behavior of a PZT bimorph homemade harvester. The Behavior Real Model proposed based on the stated space equations and the characteristic electromechanical parameters obtained experimentally has been successfully demonstrated. The advancement proposed by the present BRM model and its customized algorithms enable a piezoelectric harvesting design based on its dimensional and piezoelectric properties, where the personalization of the harvester could be achieved by the calculation of the final harvester properties based on the BRM outputs. In this way, given a specific scavenging application or source, where it is desired to extract energy, and knowing the operational frequency range, the Behavior Real Model might support the customization and optimization of the piezoelectric harvester characteristics.

Simultaneously, the electrical characteristic parameters obtained from the BRM model based on the electromechanical equivalences proposed from the Butterworth Van Dyke equivalent circuits showed a nonlinear behavior related with the dipoles charge movements and the dipoles wall movements due to the piezoelectric force caused by the sinusoidal mechanical excitation of the bimorph harvester. The understanding of the piezoelectric force, responsible for the electrical field at the harvester, and its link with the vibrational excitation is an open issue still under exploration. In the present work, some effort has been done to understand the piezoelectric force in the thickness-oriented PZT harvester; nevertheless, more experimental and theoretical research needs to be done.

Furthermore, the piezoelectric charge coefficient, *d_ij_*, behavior around the resonance frequency was analyzed based on the combined results from the BRM model and the customized iterative algorisms. The *d_ij_* allowed a better understanding of the piezoelectricity phenomenon and how it affects the energy that a harvester is able to scavenge from the applied mechanical loads. The link between the dimensional variations, the piezoelectric forces, and the piezoelectric charge coefficient is evidenced by their mechanical equivalence with the measurable equivalent characteristic parameters. In this sense, the BRM model comes close to a realistic behavior of the piezoelectric harvester and its complex nature, where the electromechanical coupling allowed a hybrid behavior from the resonance frequency due to the influence on the mechanical and dimensional properties of the piezoelectric force induced and the influence on the electrical field of the vibrational states of the mechanical characteristic parameters of the piezoelectric. This hybrid behavior redounds during the harvester constant adequation of its properties, i.e., its elongation should be adapted depending on both components, mechanical and electrical, the mechanical deformation it undergoes, and the piezoelectric force, where the BRM model, its customized iterative algorithm, and the measurable characteristic parameters proposed demonstrated their valuable potential for piezoelectric harvester understanding and design.

## Figures and Tables

**Table 1:** Equivalences between the measurable properties, electrical and mechanical, based on the Butterworth Van Dyke equivalent circuit.

Electrical Equivalent Elements	Real Measurable Properties
Electrical work (V)	Applied mechanical excitation force modulus (⌈F⌉)
Inductance, L_1_	Inertia
Resistance, R_1_	Damping
Capacitance, C_1_	1/Stiffness
N	∝ Piezoelectric charge constant
C_2_	Electrical equivalent output capacitor
R_2_	Electrical equivalent output resistance

**Table 2:** Dimensional equivalences between the electrical and mechanical properties.

Mechanical Parameters	Electrical Parameters
Mechanical force	Electrical force
N	V
Damping	Electrical resistance
$\frac{N}{m/s}$	$\frac{V}{A}=\Omega$
Stiffness	Inverse of the capacity
$\frac{N}{m}$	$\frac{V}{A\cdot s}=\frac{V}{F\cdot V}=\frac{1}{\;F}$

**Table 3:** Unit conversion according to the ratio of the piezoelectric load constant.

Primary circuit source → Integral of the current in the secondary circuit (N)·(A·s/N) → (A·s)
Secondary circuit source → Integral of the current in the primary circuit (V)·(m/V) → (m)

**Table 4:** Characteristic values, determined experimentally, for the frequency range from 15 Hz to 30 Hz at an excitation acceleration of 15 $m/s^{2}$ at the fixed (A point) and free points (B) for the BRM evaluation.

f (Hz)	$a\;\left(m/s^{2}\right)$		x (m)			$v\;\left(m/s^{2}\right)$		
	A Point	B Point	A Point	B Point	*a_fixed end_* (m/s^2^)	A Point	B Point	*a_fixed end_* (m/s^2^)
15	14.99	62.18	1.69 × 10^−3^	6.99 × 10^−3^	−14.99	9.08 × 10^−2^	0.37	12.32
18	14.95	121.39	1.17 × 10^−3^	9.47 × 10^−3^	−14.99	8.24 × 10^−2^	0.55	11.76
20	14.93	172.67	0.94 × 10^−3^	10.96 × 10^−3^	−14.90	5.82 × 10^−2^	0.91	13.10
22	14.99	257.23	0.78 × 10^−3^	13.31 × 10^−3^	−14.98	6.06 × 10^−2^	1.16	12.44
25	13	197.39	0.61 × 10^−3^	8 × 10^−3^	−14.99	8.26 × 10^−2^	1.08	7.53
30	14.27	101.54	0.40 × 10^−3^	2.85 × 10^−3^	−14.29	6.42 × 10^−2^	0.46	8.85

**Table 5:** Inductance, capacitance, and resistance of the bimorph piezoelectric harvester resulting from the experimentally measured mechanical parameters.

f (Hz)	C_1_ (F)	R_1_ (Ω)	L_1_ (H)
15	−0.05	0.32	1.79 × 10^−3^
18	−0.07	0.19	0.92 × 10^−3^
20	−0.09	0.11	0.64 × 10^−3^
22	−0.11	0.08	0.43 × 10^−3^
25	−0.07	0.06	0.49 × 10^−3^
30	−0.02	0.17	1.05 × 10^−3^

## Data Availability

Not applicable.

## References

[1] Batteries Waste Environment European Commission https://ec.europa.eu/environment/waste/batteries/.

[2] Bassi L. Industry 4.0: Hope, hype or revolution?. Proceedings of the 2017 IEEE 3rd International Forum on Research and Technologies for Society and Industry (RTSI).

[3] Perez-Alfaro I., Gil-Hernandez D., Muñoz-Navascues O., Casbas-Gimenez J., Sanchez-Catalan J.C., Murillo N. (2020). Low-Cost Piezoelectric Sensors for Time Domain Load Monitoring of Metallic Structures During Operational and Maintenance Processes. Sensors.

[4] Elvin N.G., Elvin A.A. (2013). Vibrational Energy Harvesting From Human Gait. IEEEASME Trans. Mechatron..

[5] Kishore R.A., Priya S.A. (2018). Review on Low-Grade Thermal Energy Harvesting: Materials, Methods and Devices. Materials.

[6] Sung G.-M., Chung C.-K., Lai Y.-J., Syu J.-Y. (2019). Small-Area Radiofrequency-Energy-Harvesting Integrated Circuits for Powering Wireless Sensor Networks. Sensors.

[7] Kim H.S., Kim J.-H., Kim J. (2011). A review of piezoelectric energy harvesting based on vibration. Int. J. Precis. Eng. Manuf..

[8] Madruga S., Mendoza C. (2022). Introducing a new concept for enhanced micro-energy harvesting of thermal fluctuations through the Marangoni effect. Appl. Energy.

[9] Chaari M.Z., Lahiani M., Ghariani H. Energy harvesting from electromagnetic radiation emissions by compact fluorescent lamp. Proceedings of the 2017 Ninth International Conference on Advanced Computational Intelligence (ICACI).

[10] Long Y., He P., Shao Z., Li Z., Kim H., Yao A.M., Peng Y., Xu R., Ahn C.H., Lee S.-W. (2021). Moisture-induced autonomous surface potential oscillations for energy harvesting. Nat. Commun..

[11] Shirvanimoghaddam M., Shirvanimoghaddam K., Abolhasani M.M., Farhangi M., Barsari V.Z., Liu H., Dohler M., Naebe M. (2017). Paving the Path to a Green and Self-Powered Internet of Things. arXiv.

[12] Anton S.R., Sodano H.A. (2007). A review of power harvesting using piezoelectric materials (2003–2006). Smart Mater. Struct..

[13] Lu Q., Liu L., Scarpa F., Leng J., Liu Y. (2018). A novel composite multi-layer piezoelectric energy harvester. Compos. Struct..

[14] Zhu D., Almusallam A., Beeby S., Tudor J., Harris N. A Bimorph Multi-layer Piezoelectric Vibration Energy Harvester. Proceedings of the 10th International Workshop on Micro and Nanotechnology for Power Generation and Energy Conversion Applications.

[15] Naval S., Sinha P.K., Das N.K., Anand A.K. Bandwidth Increment of Piezoelectric Energy Harvester using Multi-beam Structure. Proceedings of the 2019 Devices for Integrated Circuit (DevIC).

[16] Zhu D., Beeby S., Tudor J., White N., Harris N. (2011). Improving Output Power of Piezoelectric Energy Harvesters using Multilayer Structures. Procedia Eng..

[17] Roundy S., Wright P.K. (2004). A piezoelectric vibration based generator for wireless electronics. Smart Mater. Struct..

[18] Ma Z., Yong X. Energy harvesting characteristics of a cantilever piezoelectric transducer. Proceedings of the 2011 International Conference on Consumer Electronics, Communications and Networks (CECNet).

[19] Keawboonchuay C., Engel T.G. (2003). Maximum power generation in a piezoelectric pulse generator. IEEE Trans. Plasma Sci..

[20] Sriramdas R., Pratap R. (2018). An Experimentally Validated Lumped Circuit Model for Piezoelectric and Electrodynamic Hybrid Harvesters. IEEE Sens. J..

[21] Wu Y.-C., Halvorsen E., Lallart M., Richard C., Guyomar D. (2015). Stochastic Modeling in the Frequency Domain for Energy Harvester With Switching Electronic Interface. IEEEASME Trans. Mechatron..

[22] Ali W.G., Ibrahim S.W., Telba A. Modeling and simulation of volture piezoelectric energy harvester. Proceedings of the 2012 Seventh International Conference on Computer Engineering Systems (ICCES).

[23] Singh K.A., Kumar R., Weber R.J. (2015). A Broadband Bistable Piezoelectric Energy Harvester With Nonlinear High-Power Extraction. IEEE Trans. Power Electron..

[24] Rincón-Mora G.A., Yang S. (2016). Tiny Piezoelectric Harvesters: Principles, Constraints, and Power Conversion. IEEE Trans. Circuits Syst. Regul. Pap..

[25] Yang Y., Tang L. (2009). Equivalent Circuit Modeling of Piezoelectric Energy Harvesters. J. Intell. Mater. Syst. Struct..

[26] Kasyap A., Phipps A., Nishida T., Sheplak M., Cattafesta L. (2011). Development of MEMS-based Piezoelectric Vibration Energy Harvesters. Struct. Dyn. Renew. Energy.

[27] Rosenbaum J.F. (1988). Bulk Acoustic Wave Theory and Devices.

[28] Kim J., Varadan V.V., Varadan V.K., Bao X.-Q. (1996). Finite-element modeling of a smart cantilever plate and comparison with experiments. Smart Mater. Struct..

[29] Sun C., Shang G., Zhu X., Tao Y., Li Z. Modeling for Piezoelectric Stacks in Series and Parallel. Proceedings of the 2013 Third international conference on intelligent system design and engineering applications.

[30] Camarinha-Matos L.M., Barrento N.S., Mendonça R. Technological Innovation for Collective Awareness Systems. Proceedings of the 5th IFIP WG 5.5/SOCOLNET Doctoral Conference on Computing, Electrical and Industrial Systems, DoCEIS 2014.

[31] Liang J., Liao W. Impedance analysis for piezoelectric energy harvesting devices under displacement and force excitations. Proceedings of the 2010 IEEE International Conference on Information and Automation.

[32] Timoshenko S., Goodier J.N. (1951). Theory of Elasticity.

[33] Irwin J.D., Graf E.R. (1979). Industrial Noise and Vibration Control.

[34] Edminister J.A. (2020). Electric Circuits.

[35] Dekkers M., Boschker H., Van Zalk M., Nguyen M., Nazeer H., Houwman E., Rijnders G. (2012). The significance of the piezoelectric coefficient d31,eff determined from cantilever structures. J. Micromech. Microeng..

[36] Przybylski J., Gasiorski G. (2018). Nonlinear vibrations of elastic beam with piezoelectric actuators. J. Sound Vib..

[37] Pérez R., Albareda A., García J.E., Casals J.A. (2004). Relación entre los comportamientos no lineales dieléctrico y mecánico en cerámicas piezoeléctricas. Bol. Soc. Esp. Ceram. V..

[38] Homayouni-Amlashi A., Mohand-Ousaid A., Rakotondrabe M. (2020). Analytical modelling and optimization of a piezoelectric cantilever energy harvester with in-span attachment. Micromachines.

[39] Amerian Piezo Ceramics Inc. (2011). Piezoelectric Ceramics: Principles and Applications.

[40] Jung H.J., Jabbar H., Song Y., Sung T.H. (2016). Hybrid-type (d33 and d31) impact-based piezoelectric hydroelectric energy harvester for watt-level electrical devices. Sens. Actuators Phys..

